# The SPECTACLE Study: A Multicenter, Prospective, Single‐Arm Trial Evaluating Quantitative Blood Flow Assessment Using SPY‐QP Software in Minimally Invasive Rectal Cancer Surgery

**DOI:** 10.1111/ases.70209

**Published:** 2025-12-23

**Authors:** Atsushi Hamabe, Mamoru Uemura, Yusuke Suwa, Yujiro Nishizawa, Yoshinori Kagawa, Kei Kimura, Akihiro Kondo, Takeshi Kato, Goutaro Katsuno, Toshikatsu Nitta, Yoshinao Takano, Kinuko Nagayoshi, Shohei Miyanaga, Takeru Matsuda, Junichiro Kawamura, Jun Watanabe

**Affiliations:** ^1^ Department of Gastroenterological Surgery The University of Osaka Osaka Japan; ^2^ Department of Surgery, Gastroenterological Center Yokohama City University Medical Center Yokohama Japan; ^3^ Department of Gastroenterological Surgery Osaka General Medical Center Osaka Japan; ^4^ Department of Gastroenterological Surgery Osaka International Cancer Institute Osaka Japan; ^5^ Division of Lower GI, Department of Gastroenterological Surgery Hyogo Medical University Nishinomiya Japan; ^6^ Department of Gastroenterological Surgery, Faculty of Medicine Kagawa University Kagawa Japan; ^7^ Department of Surgery NHO Osaka National Hospital Osaka Japan; ^8^ Department of Surgery Mitsuwadai General Hospital Chiba Japan; ^9^ Division of Surgery, Gastroenterological Center Shiroyama Hospital Osaka Japan; ^10^ Department of Surgery Southern TOHOKU Research Institute for Neuroscience, Southern TOHOKU General Hospital Koriyama Japan; ^11^ Department of Surgery and Oncology Graduate School of Medical Sciences, Kyushu University Fukuoka Japan; ^12^ Department of Surgery Takaoka City Hospital Takaoka Japan; ^13^ Division of Gastrointestinal Surgery, Department of Surgery Kobe University Graduate School of Medicine Kobe Japan; ^14^ Department of Surgery Kindai University Faculty of Medicine Osaka Japan; ^15^ Department of Colorectal Surgery Kansai Medical University Hirakata Japan

**Keywords:** anastomotic leakage, fluorescence imaging, indocyanine green, rectal cancer

## Abstract

**Background:**

Anastomotic leakage (AL) remains a major postoperative complication after rectal cancer surgery, even with advances in minimally invasive techniques. Indocyanine green (ICG) fluorescence imaging is widely used to assess bowel perfusion, but conventional methods rely heavily on subjective visual interpretation. The SPY‐QP software enables quantitative evaluation of ICG fluorescence, potentially improving the accuracy of perfusion assessment.

**Methods:**

The SPECTACLE study is a multicenter, prospective, single‐arm trial designed to evaluate whether SPY‐QP‐based quantitative blood flow assessment can reduce AL rates compared with historical controls from the EssentiAL study. Perfusion assessment is performed according to an algorithm we developed based on our previous retrospective analysis. We plan to enroll 400 patients undergoing laparoscopic or robotic rectal cancer resection with anastomosis. The primary endpoint is the incidence of AL (Grades A–C) within 30 days postoperatively. Secondary endpoints include changes in surgical strategy based on perfusion findings, operative time, intraoperative complications, and other postoperative outcomes.

**Discussion:**

This study aims to provide robust evidence on whether objective perfusion assessment using SPY‐QP can reduce AL after rectal cancer surgery, potentially leading to broader adoption of quantitative imaging in colorectal surgery.

**Trial Registration:** Japan Registry of Clinical Trials: jRCTs032230212

## 1 Introduction

Anastomotic leakage (AL) following rectal cancer surgery is a serious complication that adversely affects postoperative recovery, increases healthcare costs, and compromises long‐term oncologic outcomes [1]. Despite improvements in surgical techniques and perioperative care, the incidence of AL remains unsatisfactorily high. AL is a multifactorial event, with known risk factors including male sex, low anastomosis, and advanced tumor stage [2, 3]. Adequate bowel perfusion is also critical for preventing AL, and the utility of indocyanine green (ICG) fluorescence imaging as a tool to confirm sufficient blood flow has been demonstrated in recent phase 3 trials [4, 5, 6, 7].

Although ICG fluorescence imaging has emerged as a useful adjunct for intraoperative perfusion assessment, its clinical utility is limited by the subjective interpretation of fluorescence intensity [8]. In conventional practice, evaluation depends largely on the surgeon's visual judgment, which may lead to inappropriate perfusion assessment in borderline cases and, potentially, to AL.

The SPY‐QP software system provides real‐time, quantitative measurements of parameters such as *T*_max_ (time to maximum fluorescence) and relative fluorescence intensity (RFI) compared with reference tissue. By offering objective and reproducible criteria, SPY‐QP has the potential to overcome the limitations of conventional methods. We hypothesize that quantitative assessment with SPY‐QP will improve intraoperative decision‐making regarding bowel blood flow and reduce AL rates after rectal cancer surgery.

## 2 Methods

### 2.1 Study Design

This is a multicenter, prospective, single‐arm trial conducted at tertiary referral hospitals with expertise in minimally invasive rectal cancer surgery.

### 2.2 Population

The eligibility criteria for patient enrollment were identical to those used in the EssentiAL study. Eligible patients are aged ≥ 20 years with histologically confirmed rectal cancer, defined as a tumor located within 12 cm from the anal verge. Disease must be classified as stage 0–III according to the UICC TNM 8th edition, and patients must be scheduled for curative minimally invasive surgery with an intestinal anastomosis, including procedures such as intersphincteric resection (ISR) or transanal total mesorectal excision (TaTME). The criteria for diverting stoma creation were not predefined in this study; the decision was left to the discretion of each surgeon. Additional criteria include an Eastern Cooperative Oncology Group (ECOG) performance status of 0–2, adequate organ function confirmed within 28 days prior to surgery, and written informed consent.

Patients will be excluded if they have a known allergy to iodine or ICG, require planned multivisceral resection, have unresolved bowel obstruction, require multiple colorectal anastomoses, present with an active intra‐abdominal infection, have severe uncontrolled comorbidities, or are pregnant or breastfeeding.

### 2.3 Intervention and Surgical Procedure

**FIGURE 1:**
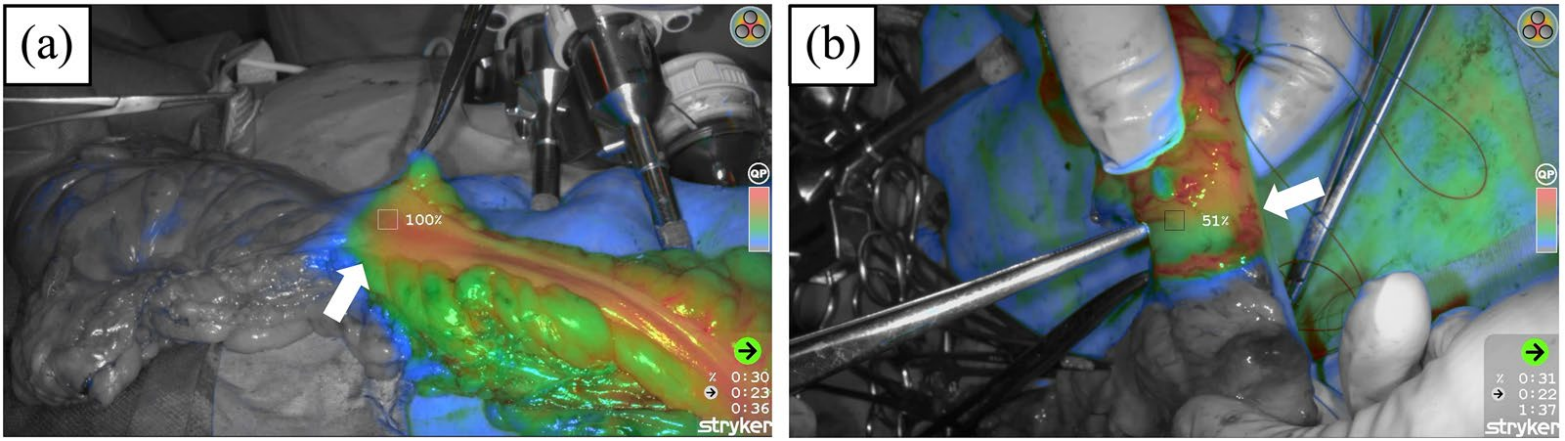
Intraoperative perfusion assessment using the SPY‐QP system. Arrows indicate the initially planned transection line determined prior to SPY‐QP evaluation after mesenteric division. (a) Case evaluated as suitable for anastomosis (time to fluorescence appearance: 25 s; *T*_max_: 23 s; relative fluorescence intensity: 100%). (b) Case determined to require additional proximal bowel resection (time to fluorescence appearance: 15 s; *T*_max_: 22 s; relative fluorescence intensity: 50%).

All procedures are performed using laparoscopic or robotic‐assisted techniques in accordance with standard oncologic principles. Following mesenteric dissection and prior to bowel division, 12.5 mg of ICG diluted in 5 mL of sterile water is injected intravenously, followed immediately by a 20 mL saline flush. Fluorescence imaging is recorded using the SPY‐PHI camera system, and perfusion is quantified with the SPY‐QP software (Figure 1). SPY‐PHI is designed to allow surgeons to visualize the operative field from a wide working distance of 20–40 cm, with fluorescence intensity remaining constant regardless of the camera‐to‐bowel distance. In this study, SPY‐PHI imaging was performed from a distance of 20–40 cm from the bowel. Because SPY‐QP enables real‐time quantification of fluorescence intensity while surgeons perform visual perfusion assessment, subjective (visual) and objective (quantitative) evaluations can be conducted simultaneously.

**FIGURE 2:**
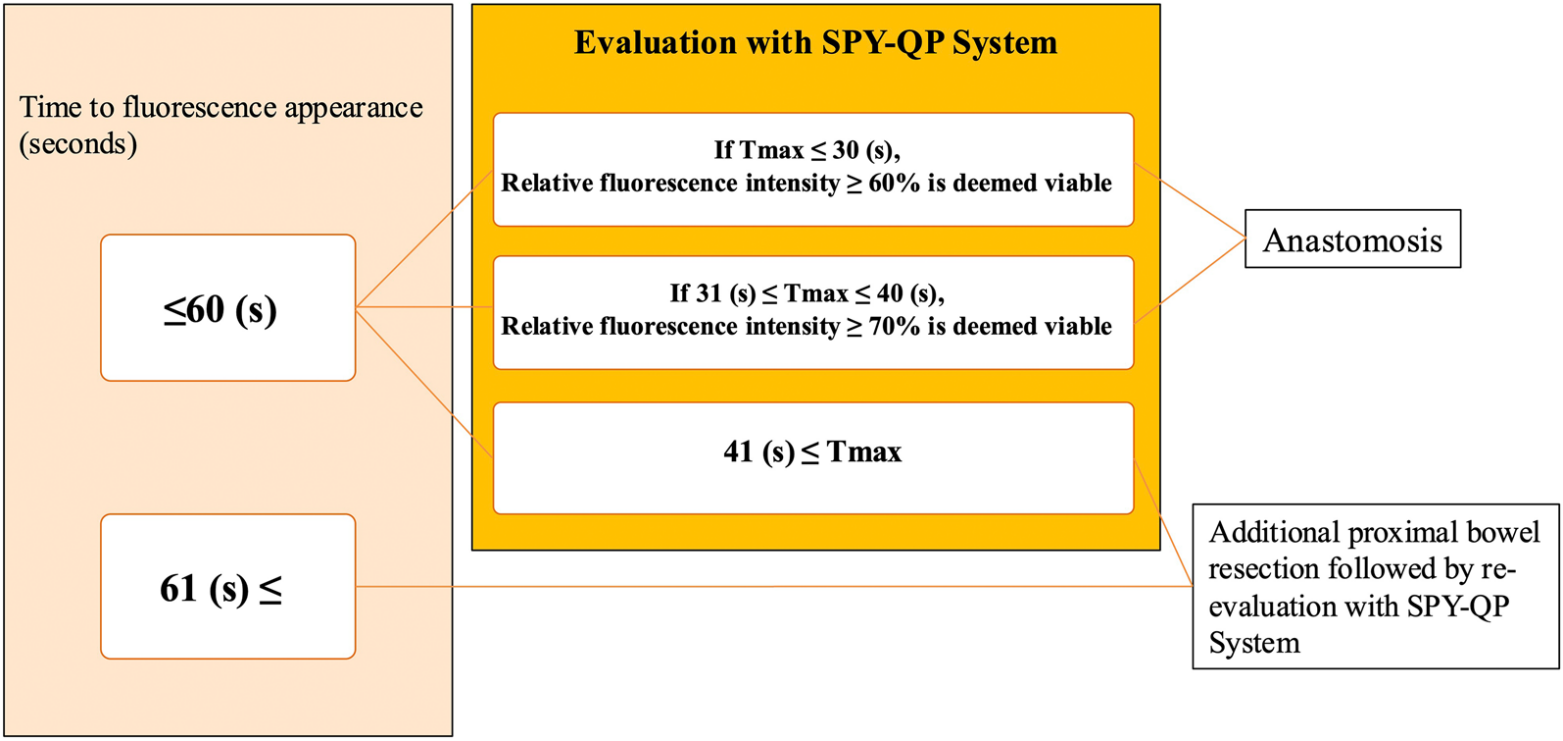
Algorithm for perfusion assessment.

Perfusion assessment is performed according to an algorithm we developed based on our previous retrospective analysis [9] (Figure 2). In our previous retrospective study, we reported that the RFI in patients without AL was ≥ 60% for *T*_max_ ≤ 30 s and ≥ 70% for *T*_max_ 31–40 s. Based on these findings, the cutoff values were determined and the algorithm was established [9]. This algorithm determines whether to proceed with gastrointestinal anastomosis or to perform additional proximal resection. Three parameters are evaluated:

- *Time to fluorescence appearance*: measured from the saline flush (0 s) to the point when fluorescence first appears in the most proximal bowel segment visible through the mini‐laparotomy.
- *T*_max_: time from fluorescence appearance to peak intensity at the reference point.
- RFI: the fluorescence intensity at the planned transection site relative to the reference point.

If the time to fluorescence appearance is ≤ 60 s, bowel transection and anastomosis are performed at a site where RFI is ≥ 60% when *T*_max_ is ≤ 30 s, or RFI is ≥ 70% when *T*_max_ is 31–40 s. If *T*_max_ is ≥ 41 s, additional proximal resection is performed, followed by reassessment with SPY‐QP. If the time to fluorescence appearance is ≥ 61 s, additional proximal resection and reassessment are also undertaken. This process is repeated until satisfactory perfusion is confirmed. Although no formal credentialing system was implemented in this study, we shared instructional videos with all participating centers and recommended that surgeons test the system in several cases before initiating patient enrollment.

### 2.4 Endpoints

#### 2.4.1 Primary Endpoint

Incidence of AL (Grades A–C) within 30 days postoperatively. To ensure accurate assessment of Grade A AL, all patients who received a diverting stoma underwent contrast enema within 30 postoperative days.

#### 2.4.2 Secondary Endpoints

- Incidence of clinically significant leakage (Grades B and C)
- Rate of resection‐line changes due to poor perfusion
- Operative time
- Blood loss
- Intraoperative adverse event rate
- Postoperative morbidity and mortality
- Length of hospital stay

### 2.5 Rationale for Sample Size

This study aimed to evaluate the superiority of SPY‐QP over a historical control in reducing the incidence of AL. In the EssentiAL study, the incidence of AL in the ICG fluorescence imaging group was 32/422 (7.6%) [5]. Assuming that SPY‐QP use would reduce this rate by 3.1% (the smallest clinically meaningful difference), the expected AL rate in this study was set at 4.5%. Using an exact binomial test, a one‐sided significance level (*α*) of ≤ 0.05 and a power (1 − *β*) of ≥ 0.8 would be achieved with a sample size of 394–400 patients. Therefore, the planned enrollment was set at 400 patients.

### 2.6 Statistical Analysis

Baseline variables will be summarized descriptively. The primary endpoint will be analyzed using a binomial test comparing observed AL rates with historical controls. Secondary endpoints will be analyzed using chi‐square tests, *t*‐tests, or nonparametric equivalents as appropriate. Missing data will be addressed using multiple imputation when applicable.

### 2.7 Data Collection and Monitoring

All data will be collected via an electronic data capture (EDC) system. Independent clinical research associates will monitor the study to ensure protocol compliance and data integrity. Patient enrollment was completed in September 2025, and the results of the primary endpoint analysis are expected to become available in the near future. The study was conducted in accordance with protocol version 4.0 (dated April 3, 2024). No substantial modifications were made to the primary endpoints or eligibility criteria across protocol versions during the study period.

### 2.8 Ethical Considerations

The protocol has been approved by the central review board at Yokohama City University. Written informed consent is obtained from all participants prior to enrollment.

## 3 Discussion

AL after rectal cancer surgery remains a significant source of postoperative morbidity and is associated with prolonged hospitalization, increased healthcare costs, and worse oncologic outcomes [3]. Although ICG fluorescence imaging has proven useful for intraoperative assessment of bowel perfusion, its impact has been limited by reliance on subjective visual interpretation. This variability in assessment is particularly problematic in borderline cases, where inadequate perfusion may not be recognized, potentially leading to AL [5].

The SPY‐QP software system provides an objective, quantitative evaluation of perfusion parameters such as *T*_max_ and RFI. By offering reproducible and standardized measurements, SPY‐QP has the potential to minimize inter‐surgeon variability in decision‐making. Objective thresholds for perfusion assessment may also facilitate clearer intraoperative judgment, particularly in cases where the clinical decision is equivocal based on visual inspection alone. We previously reported in a retrospective study that intraoperative perfusion assessment using the SPY‐QP system can quantitatively detect perfusion deficits that may be overlooked by qualitative evaluation [9]. Based on these findings, we established an algorithm for perfusion assessment using SPY‐QP.

The SPECTACLE study is designed to determine whether quantitative perfusion assessment with SPY‐QP can reduce the incidence of AL compared with historical controls from the EssentiAL study, which used conventional ICG imaging. By enrolling 400 patients across multiple high‐volume centers, this study aims to provide robust evidence regarding the clinical utility of quantitative perfusion assessment in minimally invasive rectal cancer surgery. Although ischemia‐related anastomotic stricture may occur in a small number of cases, long‐term stricture development was not included as an outcome measure in this study.

If SPY‐QP is shown to significantly reduce AL rates, the findings could support its widespread adoption as a standard adjunct in colorectal surgery. Beyond rectal cancer surgery, the technology could be applied to other gastrointestinal procedures where anastomotic integrity is critical. Ultimately, improved perfusion assessment may lead to better postoperative outcomes, reduced complication rates, and enhanced quality of life for patients.

## Author Contributions

Conceptualization, project administration, drafting of the manuscript, and writing – original draft preparation: Atsushi Hamabe, Mamoru Uemura, and Jun Watanabe. Funding acquisition and supervision: Mamoru Uemura and Jun Watanabe. Methodology: Atsushi Hamabe, Mamoru Uemura, Yusuke Suwa, Yujiro Nishizawa, Yoshinori Kagawa, Kei Kimura, Akihiro Kondo, Takeshi Kato, Goutaro Katsuno, Toshikatsu Nitta, Yoshinao Takano, Kinuko Nagayoshi, Shohei Miyanaga, Takeru Matsuda, Junichiro Kawamura, and Jun Watanabe. Writing – review and editing: Yusuke Suwa, Yujiro Nishizawa, Yoshinori Kagawa, Kei Kimura, Akihiro Kondo, Takeshi Kato, Goutaro Katsuno, Toshikatsu Nitta, Yoshinao Takano, Kinuko Nagayoshi, Shohei Miyanaga, Takeru Matsuda, and Junichiro Kawamura. All authors have read and agreed to the published version of the manuscript.

## Funding

The sponsor of the study, Stryker Japan K.K. (a subsidiary of Stryker), had no role in the study design, data gathering, analyses, interpretation, or in the writing of the manuscript or decision to submit the paper for publication, other than funding the research.

## Conflicts of Interest

Jun Watanabe reports receiving grants from Stryker Japan during the conduct of the study, and honoraria for lectures from Johnson and Johnson K.K., Covidien Japan, Eli Lilly, TERUMO, and Takeda Pharmaceutical Company Limited, and received research funding from Covidien Japan and TERUMO outside the submitted work. The other authors declare no conflicts of interest.

Junichiro Kawamura and Jun Watanabe are the Editorial Board members of *ASES* journal and the coauthors of this article. To minimize bias, they were excluded from all editorial decision‐making related to the acceptance of this article for publication.

## Data Availability

The data that support the findings of this study are available from the corresponding author upon reasonable request.
